# MAMSI: Integration of Multiassay Liquid Chromatography–Mass Spectrometry Metabolomics Data Using Multiview Machine Learning

**DOI:** 10.1021/acs.analchem.5c01327

**Published:** 2025-07-10

**Authors:** Lukas Kopecky, Caroline J. Sands, María Gómez-Romero, Shivani Misra, Elizabeth J. Want, Timothy M. D. Ebbels

**Affiliations:** † Section of Bioinformatics, Division of Systems Medicine, Department of Metabolism, Digestion and Reproduction, Faculty of Medicine, 4615 Imperial College London, London W12 0NN, U.K.; ‡ National Phenome Centre, Department of Metabolism, Digestion and Reproduction, Imperial College London, London W12 0NN, U.K.; § Metabolic Medicine, Department of Metabolism, Digestion and Reproduction, Imperial College London, London W12 0NN, U.K.; ∥ Department of Diabetes and Endocrinology, Imperial College Healthcare NHS Trust, London W12 0NN, U.K.; ⊥ Section of Bioanalytical Chemistry, Division of Systems Medicine, Department of Metabolism, Digestion and Reproduction, Faculty of Medicine, Imperial College London, London W12 0NN, U.K.

## Abstract

Liquid chromatography–mass spectrometry (LC-MS) is a commonly used analytical technique in untargeted metabolomics. However, the diverse chemical and physical properties of metabolites often require the use of several different analytical assays for broad metabolome coverage. Conventionally, each assay is analyzed separately, but this fails to capture interassay relationships, making multiassay biomarker discovery and data interpretation difficult. Here we propose a workflow to integrate multiassay metabolomics data, designed to enable biomarker discovery and elucidation of unknown metabolites. We employ a multiblock–partial least-squares model (MB-PLS) coupled with multiblock variable importance in projection to estimate the importance of predictors to the outcome variable. Then we cluster the selected predictors and compare them to groups defined by their structural properties based on retention time and mass-to-charge ratio. To demonstrate and evaluate the approach, we used three multiassay data sets predicting biological sex, Alzheimer’s disease status, and blood bilirubin levels as the outcomes of interest. The MB-PLS models outperformed single-assay models in both classification and regression tasks, indicating that modeling interblock relationships enabled an improved estimate of phenotypic outcome. Additionally, the MB-PLS models shed valuable insight into each data block’s contribution to the predicted outcome. Our workflow enabled us to determine a set of potential cross-assay biomarkers. Following putative annotation, the majority of these and their signs of association agreed with results previously reported in the literature. Our workflow has the potential to benefit the metabolomics community and beyond as it offers interpretable integrative analysis of multiassay LC-MS data and facilitates discovery of potential biomarkers.

## Graphical abstract

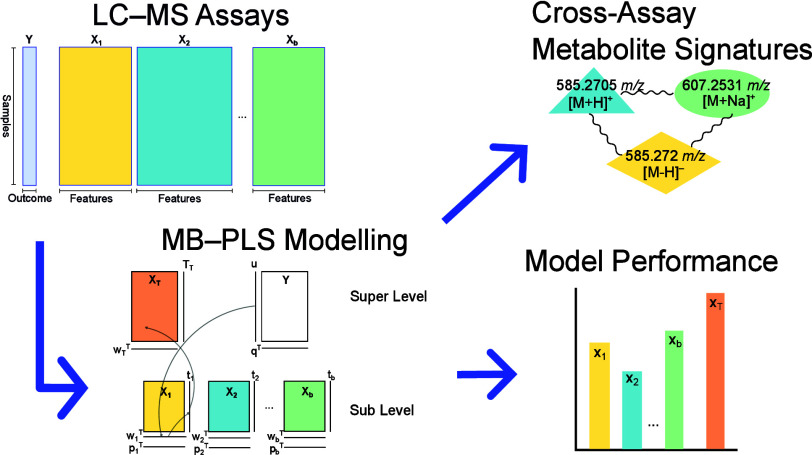

The rapid expansion of untargeted metabolomics within the past decade has been fueled by increasing demand for novel biomarkers to understand, diagnose and treat diseases.^1^ The central idea of untargeted metabolomics studies is to provide a holistic view of biological processes at the phenotypic level.^2^ Liquid chromatography–mass spectrometry (LC-MS) is one of the most popular methods for analyzing biological samples. LC-MS is a highly sensitive technique, allowing for the detection of a wide range of molecules from low-mass metabolites to lipids. However, LC-MS is unable to detect all possible molecules within a single analytical assay because of the varied physical and chemical properties of the analytes. Some molecules require positive ionization, while others require negative ionization in order to be detected. Further, molecules have different polarities, so their separation requires the use of different chromatographic columns. For example, reversed-phase chromatography (RPC) C_18_ columns are used to separate nonpolar to moderately polar compounds, whereas hydrophilic interaction chromatography (HILIC) columns are used to separate highly polar molecules.^3^ For this reason, samples are routinely processed using several analytical assays, resulting in several complementary but partially overlapping blocks of data.

A conventional approach to metabolomics data analysis is to analyze each block separately by using univariate and multivariate analysis methods. Univariate methods such as Student’s *t* tests have been used for their simplicity and reliability.^4^ However, in untargeted metabolomics data, a single metabolite can be represented by multiple features, due to processes such as in-source molecular fragmentation and isotopologue or adduct formation, whose intensities are expected to be highly correlated.^5,6^ Multivariate machine learning (ML) methods, including principal component analysis (PCA) and partial least-squares (PLS) regression, have been successfully used to exploit such correlation patterns.^4^ Despite this, there is a need for more effective methods since analyzing single assays only, even with multivariate methods, may not be sufficient to capture all the information. Some metabolites are present only in a specific assay, while other metabolites may be present in multiple assays, making interpretation a tedious process. An integrative approach would allow us to identify a comprehensible number of predictive features across these assays.

Previously, multiview (MV) ML methods have been developed to allow for the analysis of data originating from multiple sources that are related but where the exact relationship is unknown.^7^ The main objective of MV methods is to identify both the common and distinct patterns across data blocks to extract complementary information and to improve generalization of the model.^8^ An example of a MV method is the multiblock PLS (MB-PLS) model, a MV extension of PLS, that is based on the principle of maximizing the covariance between the super scores (weighted scores for each block) and the response variables.^9,10^ MB-PLS and MV models have been applied, for example, to the integration of multiomics data, or multiple spectroscopic modalities (e.g., integration of Raman spectroscopy with MS).^7^ Despite this, MB-PLS and other MV methods do not yet appear to have been used to integrate multimodal mass spectrometry metabolomics data.

It is widely known that MB-PLS has the same prediction performance as traditional PLS that uses concatenated blocks when the same feature scaling is used.^9^ However, MB-PLS allows for the application of different scalings to each block if necessary. Another advantage of using the MB approach is the additional information provided, such as block importance, making the results easier to interpret. Furthermore, compared to univariate methods that typically yield a large number of significant features, MB-PLS provides a more concise number of significant features, which can be favorable in untargeted metabolomics research, where researchers commonly deal with thousands of unannotated features.

Here, we propose the **MAMSI** (**M**ulti-**A**ssay **M**ass **S**pectrometry **I**ntegration) workflow that utilizes an MB-PLS model for integrating multiple metabolomic LC-MS assays. The main objectives of this workflow are to exploit multiassay relationships to improve the predictive power of the model and discover multiassay discriminative feature patterns. This is supported through the implementation of a tool to compare the structural relationships among statistically significant features. These comparisons help to uncover new structural information in the data that can be used to aid the search of multiassay biomarkers.

## Methods

### Data Sets

Our workflow depends on MV machine learning techniques that require numerical input in a tabular format. For this reason, the LC-MS data used in this study were preprocessed and presented in the form of feature abundance tables. To explore and validate the approach, we used data from two previously published studies run at the UK National Phenome Centre (NPC) which contain multiassay metabolomics data.

The *AddNeuroMed* study data set was collected as part of the “European Collaboration for the Discovery of Novel Biomarkers for Alzheimer’s Disease (AD)”.^11^ There are three LC-MS serum assays available: HILIC positive ionization assay (HILIC+), and lipidomic RPC positive ionization (Lipid RPC+) and negative ionization (Lipid RPC–) assays.^12^ These data were converted to mzML format using ProteoWizard,^13^ preprocessed using XCMS^14^ and nPYc toolbox^15^ software resulting in 681, 4886, and 2091 features for HILIC+, lipid RPC+, and lipid RPC– respectively. A total of 321 subjects were available: 159 AD subjects and 162 healthy controls (HC).

The *MY DIABETES* study^16^ comprises individuals of White, South Asian and African-Caribbean ancestry, mostly diagnosed with type 1 diabetes under the age of 30. Three LC-MS serum assays are available: HILIC+, Lipid RPC+ and Lipid RPC–, containing 613, 1771, and 907 features, respectively. There are also three urine assays: HILIC+, and RPC for small molecule separation in both positive ionization (SmMol RPC+) and negative ionization (SmMol RPC–) containing 2600, 12817, and 7142 features, respectively.^12^ All assays were preprocessed using ProteoWizard, XCMS and the nPYc toolbox software. A total of 984 subjects were available in this study, 540 of whom were female and 444 were male.

### MB-PLS Modeling

Multiblock partial least-squares is a multivariate regression technique used for multimodal data integration. The MB-PLS algorithm is an extension of the PLS model.^9,10^ The general form for a PLS model is

$$X=TP^{T}+E$$

$$Y=UC^{T}+G$$

where **X** is a (*N* × *M*) matrix of predictors, **Y** is a (*N* × 1) matrix of responses and **T** and **U**, both sized (*N* × *R*), are the projections (scores) of **X** and **Y** respectively onto the *R* latent variables (LVs). **P** (*M* × *R*) and **C** (1 × *R*) represent loading matrices indicating which predictors contribute to which LVs and **E** (*N* × *M*) and **G** (*N* × 1) are matrices of residuals. The decomposition of **X** and **Y** is such that the covariance between **T** and **U** is maximized. The **X**-scores are good predictors of **Y**, thus

$$Y=TC^{T}+F$$

where **F** (*N* × 1) is a further residual matrix summarizing the deviation between the observed and modeled responses. This can be expressed as a multiple regression model

Y=Xβ+F

Where **β** (*M* × 1) is a vector of regression coefficients.

MB-PLS extends this concept by introducing multiple **X** blocks (see^9^ for details). Briefly, each block **X**_**b**_ (*N* × *M*_*b*_) is used to fit a PLS model of responses **Y** to calculate the block scores **t**_**b**_ (*N* × 1). Then the block scores are combined with **Y**-scores to form the super scores **t**_**T**_ *(N* × *R)* and a PLS model between **t**_**T**_ and **Y** is fit. This is repeated for each block **X**_**1**_, . . ., **X**_**B**_. The concept is mathematically described below.

**Y**-block scores **u** are first regressed against all blocks **X**_**b**_ to calculate block variable weights **w**_**b**_ (*M*_*b*_ × 1)

$$w_{b}={\mathrm{w̃}}_{b}/∥{\mathrm{w̃}}_{b}∥$$

where

$${\mathrm{w̃}}_{b}=X_{b}^{T}u/u^{T}u$$

Block variable weights are then used to calculate block scores **t**_**b**_ (*N* × 1)

$$t_{b}=X_{b}w_{b}$$

which are collected into a matrix **T** (*N* × *B*) where

$$T=\left[t_{1},t_{2},\cdot \cdot \cdot ,t_{B}\right]$$

This is followed by calculation of superweights **w**_**T**_ (*B* × 1) obtained by regressing **u** scores against the **t**_b_

$$w_{T}={\mathrm{w̃}}_{T}/∥{\mathrm{w̃}}_{T}∥$$

where

$${\mathrm{w̃}}_{T}=T^{T}u/u^{T}u$$

Finally, super scores **t**_T_ (*N* × 1) are calculated as

$$t_{T}=Tw_{T}/w_{T}^{T}w_{T}$$

These **t**_**T**_ and **w**_**T**_ are used in PLS modeling on the super level, where components are iteratively calculated to maximize the covariance between predictors and responses.^9–17^

#### Model Building and Evaluation

We estimated the number of LVs using a grid search combined with *k*-fold cross-validation (CV). The grid search process is explained in detail in the Supporting Information (SI). To evaluate the model, we used Monte Carlo CV (MCCV), with 1000 random train-test splits, using 20% of the samples in each split for evaluation. This approach allowed us to calculate not only predictive performance using standard machine learning performance metrics but also to calculate the error margins of these metrics. However, we understand that the best estimate of confidence would come from predicting a new data set.

#### Feature Importance Estimation

We used multiblock variable importance in projection (MB-VIP)^18^ to estimate feature importance and coupled it with permutation testing to obtain empirical p-values. The mathematical definition of MB-VIP and the permutation testing procedure are given in SI.

#### Choice of Significance Level

We used a benchmarking approach to estimate the significance level for *p*-values while maintaining an interpretable number of selected features. We fitted MB-PLS models using only variables selected with different *p*-value cutoffs and compare this to the original “baseline” model (using all features). The cutoff is determined by the model performing better, or equally, compared to the baseline model while minimizing the number of significant predictors. We applied the same significance level to univariate analysis as that for the MB-PLS models. More information on this process is available in SI.

### Structural Search of Significant Features

The statistically significant MB-PLS features were clustered based on their intensity correlations. We computed a dissimilarity matrix **D** (*M* × *M*) where

$$d_{ij}=\left|1-r_{ij}\right|$$

where *r*_*ij*_ is the correlation coefficient between the *i* ^th^ and *j* ^th^ feature. We then applied agglomerative hierarchical clustering using the farthest point (complete) linkage and selected the optimal number of clusters by the highest mean silhouette score.

To investigate whether the MB-PLS derived correlation clusters are structurally related, we used retention time (*RT*) matching and mass-to-charge ratio (*m*/*z*) differences to find possible isotopologues and adducts both within and across the assays. We call these structurally linked features structural groups. The details of this structural search tool are outlined in the SI. Finally, the structural groups were compared to the correlation clusters and visualized using network plots. Additionally, our structural search tool can use region of interest (ROI) files from peakPantheR software,^19^ enabling automated annotation (minimum level 3) of features based on *m*/*z* and *RT* for a given chromatography.

### Experiments

We tested our approach in four separate *in silico* experiments using real data sets.

*Experiment 1*: We used MAMSI with MY DIABETES serum data to predict clinically measured bilirubin levels as a demonstration of a continuous outcome. Bilirubin is detectable in these assays, thus we expect to obtain bilirubin ions within the set of significant predictors.
*Experiment 2*: We applied MAMSI to explore the relationship between metabolite levels and biological sex, in MY DIABETES serum data. We used sex as it was balanced and expected to have a high effect size.
*Experiment 3*: We repeated biological sex prediction in MY DIABETES, but in urine data to examine a different sample type.
*Experiment 4*: We evaluated the workflow on a clinically relevant outcome discriminating between AD cases and HC using AddNeuroMed serum data.

## Results

### Model Performance Comparison

For all experiments, the predictive performance of the MB-PLS models was better than or comparable to that of single-block (SB) PLS models. In two out of the four experiments, the MB-PLS model appreciably outperformed the SB PLS model as demonstrated in [Table 1].

**Table 1:** Comparison of Predictive Power of SB PLS Models Compared to the MB-PLS Model Integrating All Assays^a^

	Experiment 1		Experiment 2			Experiment 3			Experiment 4		
Integrated assays	Q^2^	RMSE	Accuracy	AUC	F_1_	Accuracy	AUC	F_1_	Accuracy	AUC	F_1_
HILIC+*	0.313 ± 0.339	4.785 ± 0.230	0.872 ± 0.001	0.931 ± 0.001	0.857 ± 0.001	0.872 ± 0.001	0.912 ± 0.001	0.882 ± 0.001	0.730 ± 0.003	0.792 ± 0.003	0.716 ± 0.003
Lipid RPC+ *	0.511 ± 0.008	5.023 ± 0.057	0.813 ± 0.001	0.865 ± 0.001	0.793 ± 0.002	0.937 ± 0.001	0.951 ± 0.001	0.942 ± 0.001	0.694 ± 0.003	0.768 ± 0.003	0.682 ± 0.003
Lipid RPC– *	0.514 ± 0.008	4.989 ± 0.052	0.802 ± 0.001	0.852 ± 0.001	0.779 ± 0.002	0.943 ± 0.001	0.961 ± 0.001	0.947 ± 0.001	0.644 ± 0.003	0.706 ± 0.003	0.654 ± 0.003
MAMSI	0.603 ± 0.036	4.426 ± 0.096	0.884 ± 0.001	0.937 ± 0.001	0.871 ± 0.001	0.934 ± 0.001	0.946 ± 0.001	0.939 ± 0.001	0.724 ± 0.003	0.788 ± 0.003	0.715 ± 0.003

a The performance scores and 90% confidence intervals were calculated using MCCV with 1000 repeats. *For experiment 3, the assays are urine HILIC+, SmMol RPC+ and SmMol RPC–.

Clinically measured levels of bilirubin in blood were used as a phenotypical outcome for regression analysis in experiment 1. The increase in Q^2^ was +0.157 compared to the mean value of the SB models. At the same time the root-mean-squared error (RMSE) decreased by –0.506.

In discriminant analysis in experiment 2, the accuracy, AUC, and F_1_ scores of the integrated model increased by +0.055, +0.054, and +0.061 respectively, compared to the mean values of the SB models.

Furthermore, comparing the integrative models’ performance to the mean performance across all SB models, the integrative approach still outperforms the SB solutions. This is advantageous, especially in situations when we expect important features to be present in a worse performing block. The combination of blocks in a single MB-PLS model enabled us to increase our confidence in the data obtained from such lower performing blocks.

Another advantage of the MB-PLS model is that the model helps to identify which assays contribute most and which might be nonessential as shown in [Figure 1]. For example, for experiment 3, while the SmMol RPC+ assay had the lowest accuracy [Table 1], it provided most of the explained variance for the majority of the LVs in the model. A similar situation can be observed in experiment 4. This information would be missed if each assay were to be analyzed separately.

**Figure 1:**
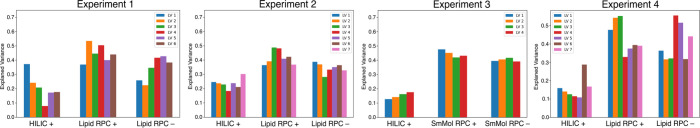
Block importance of each of the analytical assays to the predicted outcome. The bar chart shows how much of the explained variance for each latent variable (LV) in the model comes from each data block. The variance is normalized by the number of features.

### Significance Level Estimation and Comparison to Univariate Methods

The optimal significance level for *p*-values was α = 0.05 for experiment 2, α = 0.01 for experiment 4, and the Bonferroni correction for experiments 1 and 3 [SI Table 1]. The number of statistically significant predictors for the MB-PLS model was notably reduced compared to the univariate models in all four experiments [Table 2]. For experiments 2 and 3, the reduction was greater than 92%; for experiment 4, the reduction was 94%; for experiment 1, the reduction was 83%. For experiments 2 and 4, the MB-PLS model introduced additional features that did not reach significance in univariate analysis. Overall, the MB-PLS model provided a more interpretable number of features compared to univariate methods, as it is easier to annotate and explain 147 features than 2090 (Table 2).

**Table 2:** Statistically Significant Variable Comparison between Univariate Statistics and MB-PLS Models^a^

	Experiment 1 (Bonferroni)		Experiment 2 (α = 0.05)		Experiment 3 (Bonferroni)		Experiment 4 (α = 0.01)	
Assays	Univariate	MB-PLS (overlap)	Univariate	MB-PLS (overlap)	Univariate	MB-PLS (overlap)	Univariate	MB-PLS (overlap)
HILIC+*	38	10 (10)	337	51 (51)	181	13 (13)	100	5 (5)
Lipid RPC+*	43	6 (6)	1037	63 (60)	1288	79 (79)	881	40 (22)
Lipid RPC–*	45	2 (2)	626	43 (39)	621	55 (55)	247	21 (13)
Total	126	18 (18)	2000	157 (150)	2090	147 (147)	1228	66 (40)

a Features of the integrated MB-PLS model are grouped by assay of origin for clarity. For the univariate analysis, a linear model was used for experiment 1, and Mann-Whitney *U* test for experiments 2, 3, and 4. Values in brackets give the number of features from univariate tests which are significant in corresponding MB-PLS model. *For experiment 3, the assays are urine HILIC+, SmMol RPC+ and SmMol RPC–.

### Interpretation of Structural Links between Significant Features

#### Experiment 1: Prediction of Blood Bilirubin Levels

As a demonstration of a continuous outcome, we applied the MAMSI workflow to MY DIABETES serum LC-MS data to predict clinically measured bilirubin blood levels. The model yielded 18 statistically significant features that formed two structural groups and three singletons; see [Figure 2].

**Figure 2:**
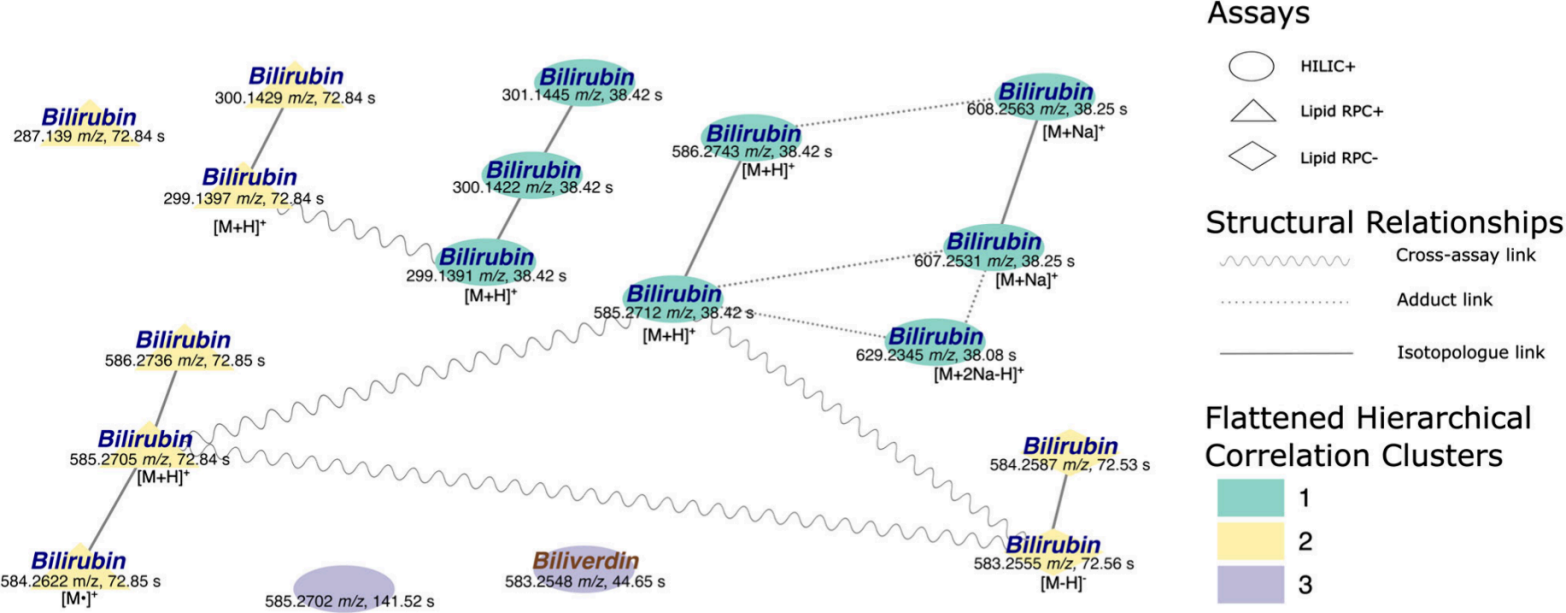
Comparison of structural relations to correlation clustering for experiment 1. Different shapes of the nodes denote analytical assays, while different colors represent different flattened hierarchical correlation clusters. Different styles of the edges represent different structural relationships between detected features. All statistically significant features for experiment 1 were included in the figure.

Using HMDB^20^ to putatively annotate the significant features, we linked 15 of these features to different bilirubin adducts, isotopologues, and fragments reported in the literature.^21,22^ Bilirubin features fell within two structural groups and one singleton. One structural group resembled isotopologue formation of protonated bilirubin’s common fragment with *m*/*z* 299.139 observed across two different assays, HILIC+ and Lipid PRC+. The other structural group linked to bilirubin (monoisotopic mass 584.2634 Da) is formed by its [M+H]^+^, [M+Na]^+^ and [M+2Na–H]^+^ adducts and an isotopologue of each of the [M+H]^+^, [M+Na]^+^ adducts, again exhibiting links across all 3 assays. All bilirubin signatures belonged to two correlation clusters, determined by the analytical assay and the ionization mode. All bilirubin signatures from the HILIC+ assay belonged to a single correlation cluster 1 (turquoise) as shown in [Figure 2]. Similarly, all bilirubin features elucidated from Lipid RPC+ and RPC– assays formed a second correlation cluster (yellow).

Part of the correlation cluster 2 was also feature 584.2622 *m*/*z*, 72.85 s *RT* which resembles the radical cation of bilirubin previously reported previously in the literature.^23^

One of the two remaining features that did not correspond to bilirubin was biliverdin, which is, from the perspective of vertebrate metabolism, an intermediate of heme catabolism where bilirubin is the end-product.^24^ This feature did not form structural groups with other features but formed correlation cluster 3 (purple) with feature 585.2702 *m*/*z*, 141.52 s *RT*.

Feature 585.2702 *m*/*z*, 141.52 s *RT* resembled an [M+H]^+^ adduct of bilirubin. However, the retention time did not match that of the bilirubin features in the HILIC+ assay. Should this feature have been bilirubin, an explanation for the disparate *RT* would be that this feature is an isomer of bilirubin as demonstrated by Albreht et al.^24^ Unfortunately, the chromatography used by Albreht et al. differs from the one used in our data set, so we were unable to annotate this feature.

Experiment 1 demonstrates that the approach can successfully model continuous outcomes. It also shows that in the relatively simple case of a single molecule outcome, ions of the same molecule are picked up successfully as important predictors, illustrating its utility in finding structurally related features.

#### Experiment 2: Prediction of Biological Sex (Serum)

Next, we applied our MAMSI workflow to predict the biological sex of the subjects in the MY DIABETES serum assays data set. We obtained 157 statistically significant features, 51 features in HILIC+, 63 from Lipid RPC+ and 43 from Lipid RPC– assays. These features formed 17 correlation clusters. 103 features were detected to form 36 structural groups; see [SI Figure 1]. Twenty-seven of these structural groups contained different isotopologues, two groups contained different adduct formations and seven groups contained both of these. Three of the structural groups were cross-assay. All structural groups agreed with correlation clusters, and their overlap is fully described in [SI Table 2].

The MAMSI workflow enabled putative annotations for 24 features. When an annotated feature was assigned to a structural group, this annotation was extended to the entire structural group. This resulted in 65 annotated features in total. Of these 24 unique annotations, 13 could be linked to metabolites reported in the literature to differentiate between sexes: proline, creatinine, betaine, cholesterol, cholesterol sulfate, phenylalanine, propionylcarnitine, isovaleryl-CoA, biliverdin, and four sphingomyelin species.^25–28^ For the full list of annotated features, see [SI Table 2].

This experiment shows that the MAMSI workflow enabled the discovery of cross-assay structural relationships that would not be possible to detect using univariate methods. Furthermore, many of those cross-assay groups match biological signatures reported in the literature.

#### Experiment 3: Prediction of Biological Sex (Urine)

We repeated the analysis of the sex prediction on MY DIABETES urine assays. In this experiment, we obtained 147 statistically significant predictors: 13 from HILC+, 79 from SmMol RPC+ and 55 in SmMol RPC–. These 147 features formed 16 correlation clusters and 1 singleton. 54 features formed 27 structural groups, see [SI Figure 2]. Of these, 10 were isotopologue groups, two were adduct groups, and five were cross-assay groups. Further, two groups contained both isotopologue and adduct links; seven groups were combinations of adduct links and cross-assay links. Finally, one group was a combination of cross-assay and adduct links.

25/27 structural groups fully agreed with correlation clustering. The other two structural groups that disagreed with correlation groups were adduct groups involving loss of water, [M+H–H_2_0]^+^ and [M–H–H_2_0]^−^.

Compared to experiment 2, the structural search provided putative annotation of only three metabolites, manifested in 15 features. Two of these, N-acetyl-L-carnosine and Dehydroisoandrosterone 3-glucuronide, were found in the SmoMol RPC– assay but formed a cross-assay group to the SmMol RPC+ assay. N-acetyl-L-carnosine could be linked to sex differences in previous literature.^26^ Experiments 2 and 3 together demonstrated that the method yields excellent results independent of the sample type analyzed. Compared to experiment 2, there is a greater cross-assay overlap.

#### Experiment 4: Prediction of Alzheimer’s Disease Status

The integrative MB-PLS model found 66 statistically significant features for discrimination between AD cases and HC in the AddNeuroMed serum data. Five of these features originated from the HILIC+, 40 from the Lipid RPC+ and 21 from the Lipid RPC– assay.

Using our structural search, we discovered 11 structural groups that included 35 features, as shown in [Figure 3]. Three structural groups contained adduct and isotopologue grouping while the remaining nine groups contained isotopologues only. Finally, there were two structural groups that contained cross-assay links. One of the structural groups linked across all three analytical assays and contained additional isotopologues in the Lipid RPC+ assay. The other cross-assay group connected HILIC+ and Lipid RPC+ only and contained isotopologue links.

**Figure 3:**
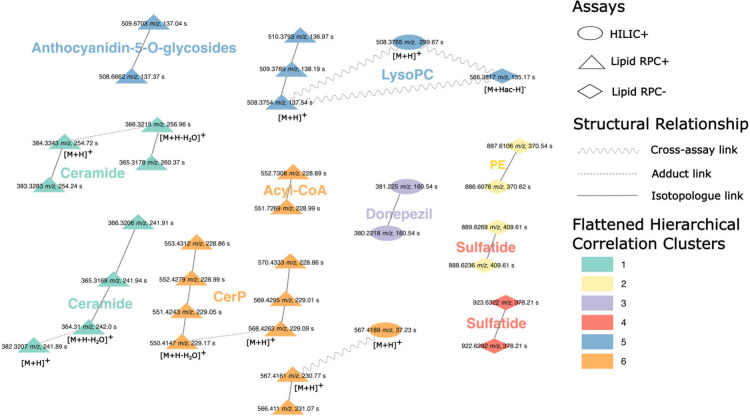
Comparison of structural relations to correlation clustering for experiment 4. Different shapes of the nodes denote that the given features originate from different analytical assays, while different colors represent belonging to a different flattened hierarchical correlation cluster. Different styles of the edges represent different structural relationships between detected features. Only features that revealed structural relationships were included in the figure.

Comparing the structural groups and correlation clusters, we found that some correlation clusters were split into multiple structural groups but all structural groups fully matched correlation clusters. This suggests that the structural links likely represent real molecular relationships and that some metabolites are correlated with each other.

Similarly to experiment 1, we employed HMDB in addition to ROI-based automated annotations, finding putative annotations for 57 features. For the full overview, see [SI Table 4]. For 42 of these features, we were able to find an association to AD in existing literature. Two features could be linked to Donepezil, a drug used to treat dementia.^29^ The remaining 40 features represented 11 distinct lipid species that belonged to four lipid classes: glycerolipids, glycerophospolipids, sphingolipids, and steroid and steroid derivatives.

Examining the effect directions, we found full literature agreement in 2/5 of molecular classes, including Donepezil, and steroids and steroid derivatives. There was a partial agreement in the remaining molecular classes depending on the particular lipids: 3/4 of sphingolipids, 3/4 of glycerophospholipids, and 1/2 of glycerolipids agreed with the literature. Where the effect direction appeared to disagree with the literature, the measured effect sizes were small and could be subject to statistical fluctuations. A full list of features associated with AD and their reported and calculated effect size is available in [SI Table 5].

With the HMDB putative annotations in *experiment 4*, we were able to compare the nature of significant features to those found in the three SB nonintegrative models. The multiblock model had an additional 16 features not present in SB models (three HILIC+, five Lipid RPC+, and eight Lipid RPC–). These included two features in HILIC+ that were both part of the two cross-assay links that would otherwise not be discovered. An overview of these features is available in [SI Table 6].

Conversely, SB models yielded 32 features not present in the MB model (five HILIC+, 19 Lipid RPC+, and eight Lipid RPC–). We were able to find putative annotations for 26 of these of which 18 were associated with AD. All putatively annotated features were of lipid classes already present in the MB model. A full overview of these features is available in [SI Table 7].

Experiment 4 illustrates that the method can predict a clinically relevant phenotype. The majority of the significant predictors matched the results reported in the literature. This indicates that the workflow is a valuable aid in the biomarker discovery process, especially when multiple assays are present.

## Discussion and Conclusion

The MAMSI workflow proved to have several advantages over conventional methods currently used in multiassay metabolomics research. The MB-PLS model can narrow the list of significant predictors by around 90% in comparison to univariate methods. This allows for an easier interpretation of the results.

A possible explanation for the reduced number of significant features in the integrated model compared to SB models is that the process of integration creates latent spaces with a greater covariance between the outcome variable and the predictors compared with LVs in SB models. For that reason, some features that were significant in the SB model are no longer significant after integration.

Further, MAMSI discovered predictors not found by the univariate approaches or in single assay PLS models, highlighting the power of multivariate and multiblock approaches to elucidate associations between metabolites and outcomes.

In our examples, the multiassay model provided better or comparable predictions of the outcome compared to models utilizing single assays. The strong predictive performance of the model plays an important role in cases where the majority of statistically significant features originate from assays that on their own have a lower predictive power compared to the integrative model. This could be observed in experiment 1 where most significant features originated from the HILIC+ assay, which on its own had a relatively low predictive performance and wide margin of error as can be seen in [Table 1]. The strong and stable performance of the MB-PLS model increases the confidence in the predictive features from the HILIC+ assay.

The comparison of structural and correlation clustering revealed that in most cases these are strongly related. This correlation structure typically has real biochemical origins. This was supported in experiment 4, predicting AD status, where putative annotations of over 64% of features matched known biomarkers of AD in the literature. Experiment 1 also reinforced this conclusion because bilirubin was both a clinically measured value and a detectable metabolite in the LC-MS serum assays. Over 89% of statistically significant features could be linked to bilirubin, its fragments, or related biliverdin, strongly supporting the utility of the MAMSI workflow as a method to find predictive multiassay features. Beyond the examples demonstrated here, our method shows promise for integration of other multimodal metabolomic data, such as different tissue/biofluid types.

Other methods have previously been published on exploiting multiple polarities in LC-MS based metabolomics, including LipidSearch^30^ and MS2DeepScore 2.0.^31^ However, these methods address the important but different problem of metabolite annotation and not the integrative analysis of multimodal metabolomics data, which is the focus of our MAMSI workflow.

While the MAMSI approach is powerful, it must be acknowledged that MB-PLS is a linear model and as such may be unable to fully capture nonlinear patterns such as detector saturation or ionization suppression effects. Additionally, visualization of results can be challenging and requires human input. Methods such as deep neural networks have the potential to solve some of these issues.^32^

In conclusion, the MAMSI workflow fills an important gap in metabolomics research as current approaches to analyze multiassay metabolomics data do not properly capture interassay connections.^7^ Our new integrative workflow utilizing the MB-PLS model exploits interassay metabolite signatures to improve prediction accuracy and interpretability of results. The MB-PLS model is an extension of traditional PLS, widely used in metabolomics research, lowering the barriers to adoption of this approach. The method outperformed univariate and SB multivariate analysis, illustrating its potential to aid in future metabolomics research as well as other disciplines that use multiassay data. The MAMSI software is freely available online as an open-source Python package.

## Supplementary Material



## Data Availability

The data sets used as part of this study are available at https://zenodo.org/records/15701829. The software developed as part of this study is available as an open-source Python package at https://pypi.org/project/mamsi/.

## Notes

Details on some methodological decisions and a detailed description of our structural search tool. Further, the SI includes network plots for experiments 2 and 3 and annotations. Further, it includes tables of annotations for experiment 4 (PDF)
