# Supplementary material for: MAMSI: Integration of Multiassay Liquid Chromatography–Mass Spectrometry Metabolomics Data Using Multiview Machine Learning
Source: Anal Chem. 2025 Jul 10;97(28):15138–45. doi: 10.1021/acs.analchem.5c01327 (PMC12291033; doi:10.1021/acs.analchem.5c01327)
Supplement: Supplementary file 1 [file ac5c01327_si_001.pdf]

Supporting Information

MAMSI: Integration of multi-assay liquid chromatography – mass spectrometry metabolomics data using multi view machine learning

Lukas Kopecky[1], Caroline J. Sands[2], María Gómez-Romero[2], Shivani Misra[3,4], Elizabeth J. Want[5], Timothy M. D. Ebbels[1*]

[1] Section of Bioinformatics, Division of Systems Medicine, Department of Metabolism, Digestion and Reproduction, Faculty of Medicine, Imperial College London, London W12 0NN, U.K.
[2] National Phenome Centre, Department of Metabolism, Digestion and Reproduction, Imperial College London, London W12 0NN, U.K.
[3] Metabolic Medicine, Department of Metabolism, Digestion and Reproduction, Imperial College London, London W12 0NN, U.K.
[4] Department of Diabetes and Endocrinology, Imperial College Healthcare NHS Trust, London W12 0NN, U.K.
[5] Section of Bioanalytical Chemistry, Division of Systems Medicine, Department of Metabolism, Digestion and Reproduction, Faculty of Medicine, Imperial College London, London W12 0NN, U.K.

Table of Contents

1. Methods *3*

1.1 Model hyperparameter estimation and performance evaluation **3**

1.2 MB-PLS Block Importance **3**

1.3 Feature importance **3**

1.4 Choice of significance level **4**

1.5 Structural search **4**

2. Results *5*

2.1 Experiment 2 ..... **6**

2.1.1 Structural to Correlation Clusters Relationship for Experiment 2: 7

2.2 Experiment 3 ..... **10**

2.3 Experiment 4 ..... **13**

2.3.1 Comparison to Non-integrative Models 19

References *23*

1. Methods

1.1 Model hyperparameter estimation and performance evaluation

The number of latent variables (LVs) in the MB-PLS model was estimated by progressively adding latent variables and using a grid search in combination with *k*-fold cross-validation. In our approach, the preferred number of LVs is the highest number for which the model performance increases by more than 5 per cent, or for which it plateaus (the performance does not change by more than 1 per cent) when adding a single LV. If these two measures do not agree, the plateau estimation is the preferred estimate. The performance of the model with the preferred number of LVs is assessed using Monte Carlo cross-validation (with 1000 random test-train splits) to prevent overfitting.

1.2 MB-PLS Block Importance

Block importance for a latent variable r and block b can be calculated as

$$BIP_{rb} = (w_{rb})^2$$

However, block importance depends on the number of features p in each block, we use corrected BIP [1] which can be calculated as

$$BIP_{corr,rb} = BIP_{rb} \cdot (1 - \frac{p_b}{p_{total}}).$$

1.3 Feature importance

The Variable importance in projection (VIP) is based on the PLS weights weighted by the proportion of explained variance in **Y** for each LV normalized by the sum of squares across all LVs in the model [2]. Weider et al. [3] proposed a multi-block extension of this method called multiblock VIP (MB-VIP). The MB-VIP for each feature can be calculated as

$$\text{MB-VIP}_j = \sqrt{\frac{f \cdot \sum_{r=1}^{\mathrm{R}} (w_{krj}^2 \cdot \mathbf{SSY}_r)}{\mathbf{SSY}_{\mathrm{cum}}}}$$

where the w_{krj} is the weight for j^{th} feature in k^{th} block and r^{th} LV. $\mathbf{SSY}_r$ is the sum of squares of explained **Y** variance and f is the number of features across all **X** blocks. $\mathbf{SSY}_{cum}$ is the total sum of squares of **Y**. We coupled this method with permutation testing to estimate empirical p-values for each variable. This was done by randomly permuting the Y outcome variable 1 million times and refitting the model; the empirical p-values for each feature were then calculated by counting the number of trials with MB-VIP greater than or equal to the observed test statistic, and dividing this by 1 million [3].

1.4 Choice of significance level

A conventional approach of interpretating empirical p-values would be to control for Type 1 errors by applying a multiple-testing correction method. However, individual features in LC-MS data do not necessarily represent different molecular species. Applying too conservative an approach, such as Bonferroni correction, could lead to loss of valuable information in the biological interpretation of the results. Furthermore, increased false positive rate is not a crucial issue in untargeted metabolomics due to the focus on discovery and hypothesis generation. Using too strict a threshold will limit power and lead to a large number of false negatives. For that reason, we used a benchmarking approach (described in main text) to determine the desired p-value cut-off while maintaining an interpretable number of selected features.

1.5 Structural search

Our structural search tool is based on an adduct calculator developed by Tobias Kind at the Fiehn Lab, UC Davies [4]. First, all features are split into retention time (*RT*) windows of 5 s intervals, then each *RT* window is searched for ^{12}C-^{13}C isotopologue signatures by searching mass differences of 1.00335 Da between features; if two or more features fit an isotopologue signature then they are grouped together. This is followed by a search for common adduct signatures. This is achieved by calculating hypothetical neutral masses based on common adducts in electrospray ionization [5]. If hypothetical neutral masses match for two or more features within a pre-defined tolerance (15 ppm) then these features are grouped together. Overlapping adduct, and isotopologue clusters are then merged to form structural clusters. Further, MAMSI automatically searches for cross-assay clusters using $[M+H]^+$ to $[M-H]^-$ relationships. We then expanded this by searching for different cross-assay adduct relationships manually.

Additionally, our structural search tool, that utilizes region of interest (ROI) files [6] from peakPantheR [7], allows for automated annotation of some features based on the *RT* for a given chromatography and *m/z*.

2. Results

Table 1. Cut-off estimation for statistical significance. The table compares the performance of the baseline model utilizing all features to models utilizing only statistically significant variables with different cut-offs of significance and multiple testing correction, including false discovery rate (FDR) and Bonferroni (Bonf.) correction. The performance scores (mean and 90% confidence interval) were measured using Monte Carlo cross-validation (MCCV) with 1000 repeats. Selected cut-off highlighted in bold.

Cut-off	*Experiment 1*		*Experiment 2*				*Experiment 3*				*Experiment 4*			
	Q^2	*MSE*	*ACC*	*AUC*	*Recall*	F_1	*ACC*	*AUC*	*Recall*	F_1	*ACC*	*AUC*	*Recall*	F_1
Baseline	0.603 ±0.036	4.426 ±0.096	0.884 ±0.001	0.937 ±0.001	0.879 ±0.002	0.871 ±0.001	0.934 ±0.001	0.946 ±0.001	0.948 ±0.001	0.939 ±0.001	0.724 ±0.003	0.788 ±0.003	0.704 ±0.004	0.715 ±0.003
a=0.05	0.660 ±0.008	4.196 ±0.066	**0.880 ±0.001**	**0.940 ±0.001**	**0.862 ±0.002**	**0.865 ±0.001**	0.955 ±0.001	0.986 ±0.000	0.972 ±0.001	0.959 ±0.001	0.864 ±0.002	0.945 ±0.001	0.839 ±0.003	0.858 ±0.002
a=0.01	0.639 ±0.008	4.330 ±0.067	0.862 ±0.001	0.929 ±0.001	0.843 ±0.002	0.845 ±0.001	0.959 ±0.001	0.986 ±0.000	0.980 ±0.001	0.963 ±0.001	**0.785 ±0.003**	**0.874 ±0.002**	**0.724 ±0.004**	**0.767 ±0.003**
FDR	0.701 ±0.007	3.897 ±0.064	0.738 ±0.002	0.830 ±0.001	0.585 ±0.003	0.666 ±0.002	0.961 ±0.001	0.983 ±0.001	0.983 ±0.001	0.957 ±0.001	0.737 ±0.003	0.823 ±0.002	0.541 ±0.004	0.667 ±0.004
Bonf.	**0.699 ±0.008**	**3.909 ±0.064**	0.736 ±0.002	0.823 ±0.001	0.575 ±0.003	0.661 ±0.002	**0.941 ±0.001**	**0.982 ±0.001**	**0.985 ±0.001**	**0.948 ±0.001**	0.740 ±0.002	0.800 ±0.003	0.547 ±0.004	0.673 ±0.003

2.1 Experiment 2

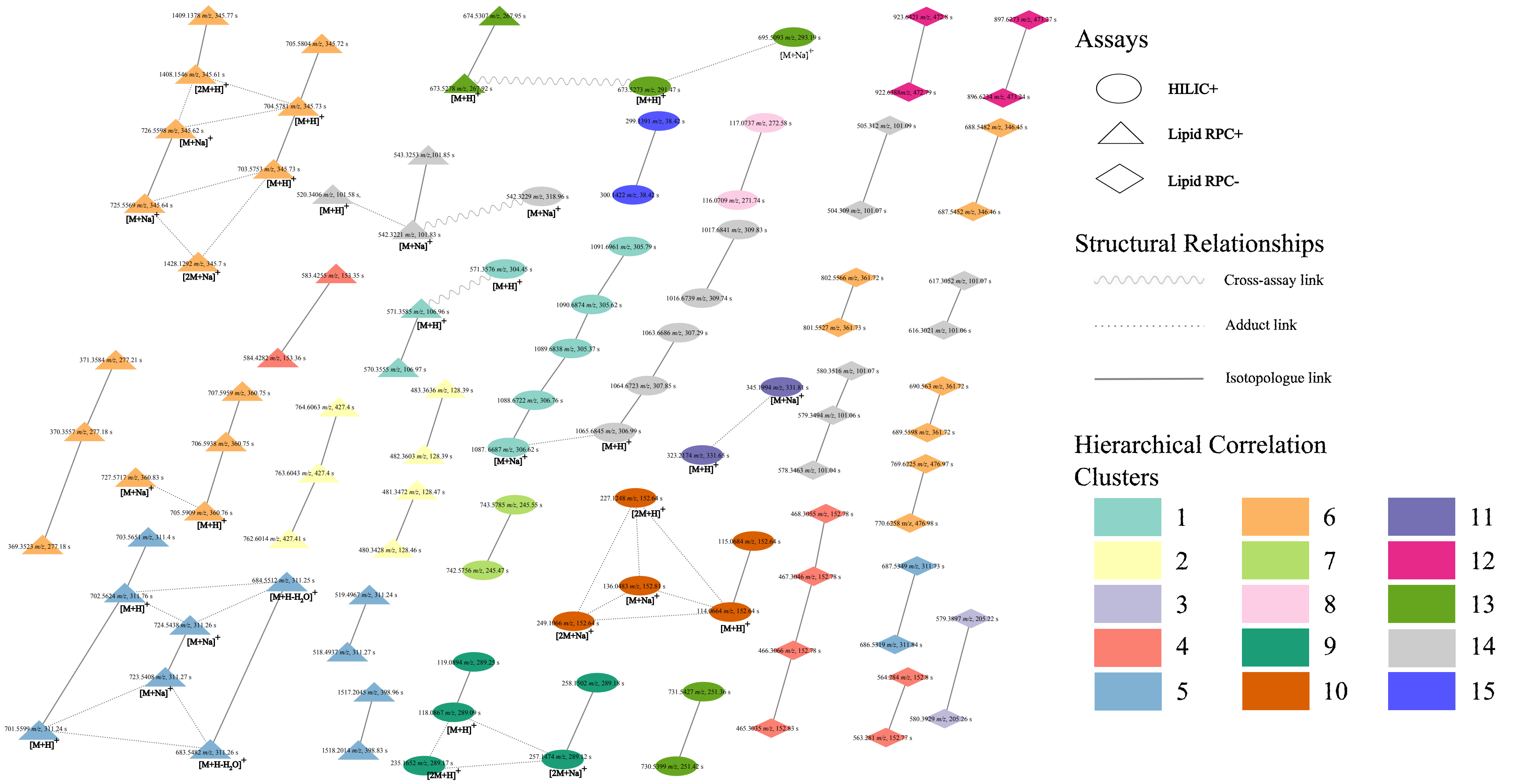

Figure 1. Comparison of structural relations to correlation clustering for experiment 2. Different shapes of the nodes denote different analytical assays, while different colors represent different flattened hierarchical correlation clusters. Different styles of the edges represent different structural relationship between detected features. Only features with structural relationships were included.

2.1.1 Structural to Correlation Clusters Relationship for Experiment 2:

There were 3 cross-assay structural links in the data; one of those connected structural clusters while the other two connected a structural cluster in one assay and a single feature in the other, see [SI Figure 1]. All structural clusters and cross-assay clusters agreed with correlation clustering except one structural cluster which was split between two correlation clusters (1 turquoise and 14 gray). Seven correlation clusters contained only one structural cluster, while 7 correlation clusters contained between 2 to 5 structural clusters each, and 1 correlation cluster contained 7 structural clusters. One correlation cluster did not contain any structural clusters but only structurally unrelated variables (not present in [SI Figure 1] as this contains only structural relationships).

Table 2. Table of putative annotations for experiment 2 features. Column 'Annotation Kind' indicates whether the annotation was automatically obtained using peakPantheR Region of Interest (ROI) files in MAMSI (minimum level 3 annotation) denoted as 'ROI', or whether it was found by extending a ROI annotation to the entire structural cluster (level 3 annotation) denoted as 'extended'. The 'Structural cluster' column is a combination of 'Isotopologue group' and 'Adduct Group' columns. The 'Cross-assay Link' column shows the features that connect a structural cluster across different assays. Only annotated features are present.

Assay	*RT (sec)*	*m/z*	*Isotopologue group*	*Isotopologue pattern*	*Adduct group*	*Adduct*	*Structural cluster*	*Cross-assay link*	*Annotation kind*	*Annotation*
HILIC +	271.74	116.0709	2	M			1		ROI	Proline
HILIC +	272.58	117.0737	2	M+1			1		Extended	Proline
HILIC +	152.64	114.0664	1	M	1	$[M+H]^+$	2		Extended	Creatinine
HILIC +	152.64	115.0684	1	M+1			2		Extended	Creatinine
HILIC +	152.81	136.0483			1	$[M+Na]^+$	2		ROI	Creatinine
HILIC +	152.64	227.1248			1	$[2M+H]^+$	2		Extended	Creatinine
HILIC +	152.64	249.1066			1	$[2M+Na]^+$	2		Extended	Creatinine
HILIC +	289.09	118.0867	3	M	2	$[M+H]^+$	3		ROI	Betaine
HILIC +	289.25	119.0894	3	M+1			3		Extended	Betaine
HILIC +	289.12	257.1474	4	M	2	$[2M+Na]^+$	3		Extended	Betaine
HILIC +	289.18	258.1502	4	M+1			3		Extended	Betaine
HILIC +	289.17	235.1652			2	$[2M+H]^+$	3		Extended	Betaine
HILIC +	291.47	673.5273			3	$[M+H]^+$	4	4	Extended	SM(d18:2/14:0)

Lipid RPC +	267.92	673.5278	9	M			4	4	ROI	SM(d18:2/14:0)
HILIC +	293.19	695.5093			3	$[M+Na]^+$	4		Extended	SM(d18:2/14:0)
Lipid RPC +	267.95	674.5307	9	M+1			4		Extended	SM(d18:2/14:0)
Lipid RPC +	277.18	369.3523	5	M			5		ROI	Cholesterol
Lipid RPC +	277.18	370.3557	5	M+1			5		Extended	Cholesterol
Lipid RPC +	277.21	371.3584	5	M+2			5		Extended	Cholesterol
Lipid RPC +	128.46	480.3428	6	M			6		ROI	LPC(P-16:0/0:0)
Lipid RPC +	128.47	481.3472	6	M+1			6		Extended	LPC(P-16:0/0:0)
Lipid RPC +	128.39	482.3603	7	M			7		ROI	LPC(O-16:0/0:0)
Lipid RPC +	128.39	483.3636	7	M+1			7		Extended	LPC(O-16:0/0:0)
Lipid RPC +	106.96	571.3585	8	M+1		$[M+H]^+$	8	8	Extended	LPC(0:0/22:5)_1
Lipid RPC +	106.97	570.3555	8	M			8		ROI	LPC(0:0/22:5)_1
Lipid RPC +	427.41	762.6014	16	M			9		ROI	PC(34:0); PC(18:0/16:0) | PC(16:0/18:0)
Lipid RPC +	427.4	763.6043	16	M+1			9		Extended	PC(34:0); PC(18:0/16:0) | PC(16:0/18:0)
Lipid RPC +	427.4	764.6063	16	M+2			9		Extended	PC(34:0); PC(18:0/16:0) | PC(16:0/18:0)
Lipid RPC +	101.83	542.3221	15	1	4	$[M+Na]^+$	10	10	Extended	LPC(0:0/18:2)
HILIC +	318.96	542.3229				$[M+Na]^+$	10	10	Extended	LPC(0:0/18:2)
Lipid RPC +	101.58	520.3406			4	$[M+H]^+$	10		ROI	LPC(0:0/18:2)
Lipid RPC +	101.85	543.3253	15	2			10		Extended	LPC(0:0/18:2)
Lipid RPC +	311.26	683.5482	10	M	5	$[M+H-H_2O]^+$	11		Extended	SM(d18:2/16:0)_1
Lipid RPC +	311.25	684.5512	10	M+1	6	$[M+H-H_2O]^+$	11		Extended	SM(d18:2/16:0)_1
Lipid RPC +	311.24	701.5599	11	M	5	$[M+H]^+$	11		ROI	SM(d18:2/16:0)_1

Lipid RPC +	311.76	702.5624	11	M+1	6	[M+H]+	11		Extended	SM(d18:2/16:0)_1
Lipid RPC +	311.4	703.5651	11	M+2			11		Extended	SM(d18:2/16:0)_1
Lipid RPC +	311.27	723.5408	14	M	5	[M+Na]+	11		Extended	SM(d18:2/16:0)_1
Lipid RPC +	311.26	724.5438	14	M+1	6	[M+Na]+	11		Extended	SM(d18:2/16:0)_1
Lipid RPC +	345.73	703.5753	12	M	7	[M+H]+	12		ROI	SM(d18:1/16:0)
Lipid RPC +	345.73	704.5781	12	M+1	8	[M+H]+	12		Extended	SM(d18:1/16:0)
Lipid RPC +	345.72	705.5804	12	M+2			12		ROI	SM(d18:0/16:0)
Lipid RPC +	345.64	725.5569	15	M	7	[M+Na]+	12		Extended	SM(d18:1/16:0)
Lipid RPC +	345.62	726.5598	15	M+1	8	[M+Na]+	12		Extended	SM(d18:1/16:0)
Lipid RPC +	345.61	1408.155	17	M	8	[2M+H]+	12		Extended	SM(d18:1/16:0)
Lipid RPC +	345.77	1409.138	17	M+1			12		Extended	SM(d18:1/16:0)
Lipid RPC +	345.7	1428.129			7	[2M+Na]+	12		Extended	SM(d18:1/16:0)
Lipid RPC +	360.76	705.5909	13	M	9	[M+H]+	13		ROI	SM(d18:0/16:0)
Lipid RPC +	360.75	706.5938	13	M+1			13		Extended	SM(d18:0/16:0)
Lipid RPC +	360.75	707.5959	13	M+2			13		Extended	SM(d18:0/16:0)
Lipid RPC +	360.83	727.5717			9	[M+Na]+	13		Extended	SM(d18:0/16:0)
Lipid RPC -	152.83	465.3035	18	M			14		ROI	Cholesterol Sulfate_1
Lipid RPC -	152.78	466.3066	18	M+1			14		Extended	Cholesterol Sulfate_1
Lipid RPC -	152.78	467.3046	18	M+2			14		Extended	Cholesterol Sulfate_1
Lipid RPC -	152.78	468.3055	18	M+3			14		Extended	Cholesterol Sulfate_1
HILIC +	304.45	571.3576				[M+H]+	8	8	Extended	LPC(0:0/22:5)_1
HILIC +	44.65	583.2548							ROI	Biliverdin
Lipid RPC +	314.39	540.4984							ROI	Hexacosanoylcarnitine CAR(26:0)
HILIC +	275.65	246.1699							ROI	Isovaleryl | valeryl |

										2-methylbutyryl carnitine CAR(5:0)
Lipid RPC +	162.76	510.3915							ROI	LPC(O-18:0/0:0)
Lipid RPC +	162.68	508.376							ROI	LPC(P-18:0/0:0)
HILIC +	238.13	166.0863							ROI	Phenylalanine
HILIC +	297.76	218.1389							ROI	Propionylcarnitine CAR(3:0)
Lipid RPC +	400.23	757.6222							ROI	SM(d18:1/20:1)
Lipid RPC +	317.82	701.5592							ROI	SM(d18:2/16:0)_1

2.2 Experiment 3

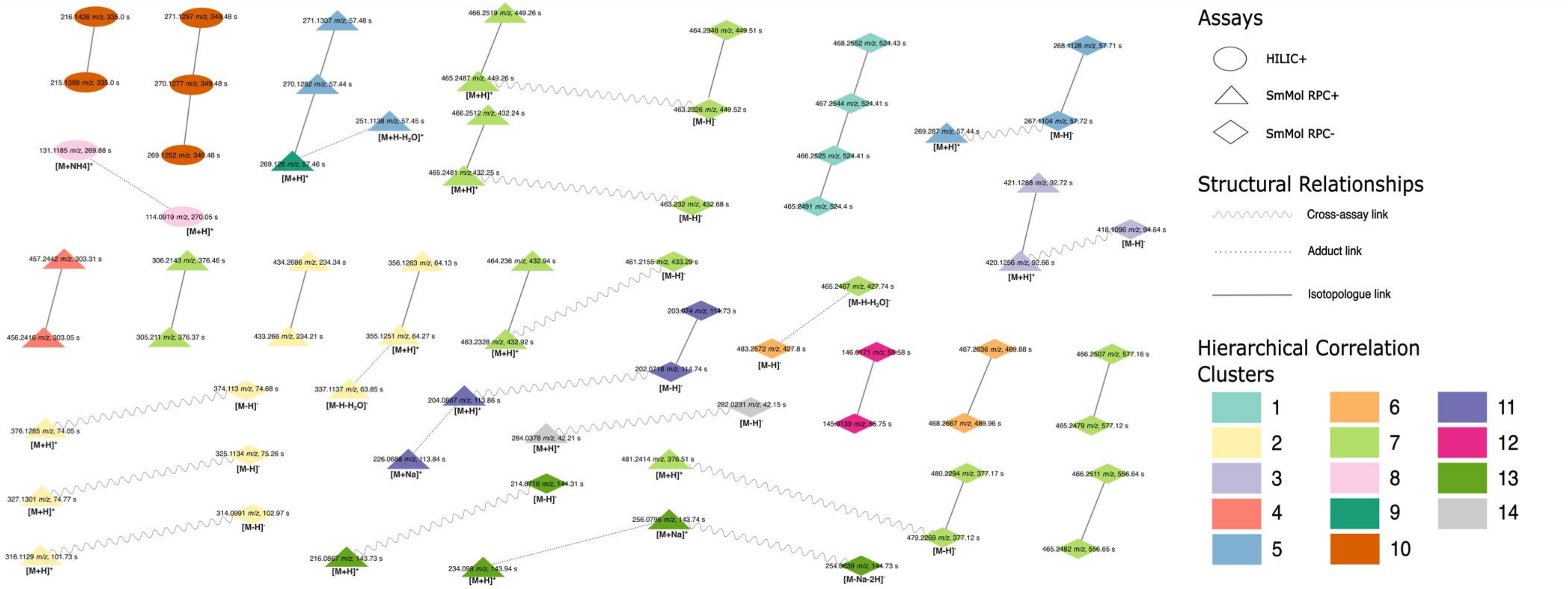

Figure 2. Comparison of structural relations to correlation clustering for experiment 3. Different shapes of the nodes denote different analytical assays, while different colors represent different flattened hierarchical correlation clusters. Different styles of the edges represent different structural relationships between detected features. Only features with structural relationships were included.

Table 3. Table of putative annotations for experiment 3 features. Column 'Annotation Kind' indicates whether the annotation was automatically obtained using peakPantheR Region of Interest (ROI) files in MAMSI (minimum level 3 annotation) denoted as 'ROI', or whether it was found by extending a ROI annotation to the entire structural cluster (level 3 annotation) denoted as 'extended'. The 'Structural cluster' column is a combination of 'Isotopologue group' and 'Adduct Group' columns. The 'Cross-assay Link' column shows the features that connect a structural cluster across different assays. Only annotated features are present.

Assay	*RT (sec)*	*m/z*	*Isotopologue group*	*Isotopologue pattern*	*Adduct group*	*Adduct*	*Structural cluster*	*Cross-assay link*	*Annotation kind*	*Putative Annotation*
HILIC +	270.1	114.0919			1	[M+H]+	1		Extended	N-Acetylputrescine
HILIC +	269.9	131.1185			1	[M+NH4]+	1		ROI	N-Acetylputrescine
SmMol RPC +	432.3	465.2481	1	M			2	2	Extended	Dehydroisoandrosterone 3-glucuronide
SmMol RPC +	432.2	466.2512	1	M+1			2		Extended	Dehydroisoandrosterone 3-glucuronide
HILIC +	349.5	269.1252	2	M			2		Extended	Dehydroisoandrosterone 3-glucuronide
HILIC +	349.5	270.1277	2	M+1			2		Extended	Dehydroisoandrosterone 3-glucuronide
HILIC +	349.5	271.1297	2	M+2			2		Extended	Dehydroisoandrosterone 3-glucuronide
SmMol RPC -	432.7	463.232					2	2	ROI	Dehydroisoandrosterone 3-glucuronide
SmMol RPC +	449.3	465.2487	3	M			3	3	Extended	Dehydroisoandrosterone 3-glucuronide
SmMol RPC +	449.3	466.2519	3	M+1			3		Extended	Dehydroisoandrosterone 3-glucuronide
SmMol RPC -	449.5	463.2326	5	M			3	3	ROI	Dehydroisoandrosterone 3-glucuronide

SmMol RPC -	449.5	464.2346	5	M+1			3		Extended	Dehydroisoandrosterone 3-glucuronide
SmMol RPC -	57.72	267.1104	4	M			4	4	ROI	N-acetyl-L-carnosine
SmMol RPC -	57.71	268.1128	4	M+1			4		Extended	N-acetyl-L-carnosine
SmMol RPC +	57.44	269.287					4	4	Extended	N-acetyl-L-carnosine

2.3 Experiment 4

Table 4. Table of putative annotations for experiment 4 features. Column 'Annotation Kind' indicates whether the annotation was automatically obtained using peakPantheR Region of Interest (ROI) files in MAMSI (minimum level 3 annotation) denoted as 'ROI', features annotated using HMDB (level 3) denoted as 'HMDB', or whether it was found by extending a ROI/HMDB annotation to the entire structural cluster (level 3 annotation) denoted as 'extended'. The 'Structural cluster' column is a combination of 'Isotopologue group' and 'Adduct Group' columns. The 'Cross-assay Link' column shows the features that connect a structural cluster across different assays. Only annotated features are present.

Assay	*RT (sec)*	*m/z*	*Isotopologue group*	*Isotopologue pattern*	*Adduct group*	*Adduct*	*Structural cluster*	*Cross-assay link*	*Annotation Kind*	*Putative Annotation*
HILIC +	160.54	380.2218	1	M			1		HMDB	Donepezil
HILIC +	160.54	381.225	1	M+1			1		HMDB	Donepezil
Lipid RPC +	137.54	508.3754	5	M			2	2	HMDB | ROI	LPC(O-18:1/0:0)_1
Lipid RPC +	138.19	509.3789	5	M+1			2		Extended	LPC(O-18:1/0:0)_1
Lipid RPC +	136.97	510.3793	5	M+2			2		Extended	LPC(O-18:1/0:0)_1
Lipid RPC +	137.37	508.6662	6	M			3		HMDB	Anthocyanidin-5-O-glycosides
Lipid RPC +	137.04	509.6703	6	M+1			3		Extended	Anthocyanidin-5-O-glycosides
Lipid RPC +	228.99	551.7269	8	M			4		HMDB	acyl-CoA

Lipid RPC +	228.89	552.7308	8	M+1			4		Extended	acyl-CoA
Lipid RPC +	242	364.31	2	M	1	$[M+H-H_2O]^+$ | $[M+3ACN-2H]^+$	5		HMDB	Cer(20:1/18:3-OH)
Lipid RPC +	241.94	365.3169	2	M+1			5		Extended	Cer(20:1/18:3-OH)
Lipid RPC +	241.91	366.3206	2	M+2			5		Extended	Cer(20:1/18:3-OH)
Lipid RPC +	241.89	382.3207			1	$[M+H]^+$ | $[M+3ACN-2H+H_2O]+$	5		Extended	Cer(20:1/18:3-OH)
Lipid RPC +	260.37	365.3178	3	M			6		HMDB	Cer(20:1/18:1-O)
Lipid RPC +	256.96	366.3215	3	M+1	2	$[M+H-H_2O]^+$ | $[M+3ACN-2H]^+$	6		Extended	Cer(20:1/18:1-O)
Lipid RPC +	254.24	383.3283	4	M			6		Extended	Cer(20:1/18:1-O)
Lipid RPC +	254.72	384.3343	4	M+1	2	$[M+H]^+$ | $[M+3ACN-2H+H_2O]+$	6		Extended	Cer(20:1/18:1-O)
Lipid RPC +	229.05	551.4243	7	M+1			7		Extended	CerP(18:1/12:0)
Lipid RPC +	228.99	552.4279	7	M+2			7		Extended	CerP(18:1/12:0)
Lipid RPC +	228.86	553.4312	7	M+3			7		Extended	CerP(18:1/12:0)
Lipid RPC +	229.09	568.4263	9	M	3	$[M+H]^+$ | $[M+3ACN-2H+H_2O]+$	7		HMDB	CerP(18:1/12:0)
Lipid RPC +	229.01	569.4295	9	M+1			7		Extended	CerP(18:1/12:0)
Lipid RPC +	228.86	570.4333	9	M+2			7		Extended	CerP(18:1/12:0)
Lipid RPC -	370.62	886.6076	10	M			8		HMDB	PE(24:1/20:3)
Lipid RPC -	370.54	887.6106	10	M+1			8		Extended	PE(24:1/20:3)

Lipid RPC -	409.61	888.6236	11	M			9		HMDB	3-O-Sulfogalactosylceramide (d18:1/24:1)
Lipid RPC -	409.61	889.6269	11	M+1			9		Extended	3-O-Sulfogalactosylceramide (d18:1/24:1)
Lipid RPC -	378.21	922.6292	12	M			10		HMDB	3-O-Sulfogalactosylceramide (d18:1/22:0)
Lipid RPC -	378.21	923.6322	12	M+1			10		Extended	3-O-Sulfogalactosylceramide (d18:1/22:0)
Lipid RPC +	229.17	550.4147	7	M	3	$[M+H-H_2O]^+$ | $[M+3ACN-2H]^+$	7		Extended	CerP(d18:1/12:0)
HILIC +	299.67	508.3765					2	2	HMDB | ROI	LPC(O-18:1/0:0)_1
Lipid RPC -	135.17	566.3817					2	2	Extended	LPC(O-18:1/0:0)_1
Lipid RPC -	275.33	551.4665							HMDB	1-Triacontanol
Lipid RPC -	198.15	539.4307							HMDB	2-Hydroxydodecyl methacrylate
Lipid RPC -	173.46	538.4185							HMDB	25-Hydroxyprotopanaxadiol
Lipid RPC -	371.72	860.5923							HMDB | ROI	3-O-Sulfogalactosylceramide (18:1/22:0) | SulfoHexCer(18:2/22:0)
Lipid RPC -	75.03	481.2983							HMDB	5-Cholesten-3beta-25-diol-3-sulfate

Lipid RPC +	228.84	568.7335							HMDB	CDP-DG(20:4/PGD2)
Lipid RPC +	256.55	365.5645							HMDB	CDP-DG(i-24:0/i-18:0)
Lipid RPC +	496.16	667.6431							HMDB	CE(19:0)
Lipid RPC +	216.5	363.3022							HMDB	Cer(18:2/20:3-OH)
Lipid RPC -	253.87	437.3621							HMDB	DG(16:0/8:0/0:0)
Lipid RPC +	524.52	831.7214							HMDB	DG(24:0/24:0/0:0)
Lipid RPC +	136.99	508.6063							HMDB	Dodec-6-enedioyl-CoA
Lipid RPC +	260.67	569.4292							HMDB	LysoPC(20:0/0:0)
HILIC +	138.81	470.2318							HMDB	Nefazodone
Lipid RPC +	228.85	459.3588							HMDB	PC(O-22:3/22:3)
Lipid RPC +	256.42	381.3129							HMDB	Nymphayol
Lipid RPC -	331.81	832.5605							HMDB	PE(20:3-OH/22:4)
Lipid RPC -	401.16	904.6181							HMDB	PE(20:3-OH/24:0)
Lipid RPC -	361.37	903.6056							HMDB	PE(20:3-OH/24:1)
Lipid RPC -	421.38	830.642							HMDB	PE(P-18:1/24:)
Lipid RPC +	462.05	776.0123							HMDB	Phosalone
Lipid RPC -	305.95	867.5388							HMDB	PI(18:0/20:4)
Lipid RPC +	477.73	769.6587							HMDB	SM(18:1/22:0)
Lipid RPC +	493.85	825.6756							HMDB	TG(15:0/20:4/18:4)
Lipid RPC -	139.83	463.2883							HMDB	5-Cholesten-3beta-25-diol-3-sulfate

Table 5. Table of MB-PLS features associated with AD literature. The 'Expected Effect Direction' column was extracted from literature as reported in 'PMID' column. PMID values are highlighted in bold signify that the PMID was provided via corresponding HMDB, otherwise the citation comes from manual literature search. Cliff's delta (CD) calculation was done based on LC-MS profiles. Positive CD values signify that the metabolite abundance was increased in AD group, negative CD values mean that metabolite abundance was increased in healthy controls. The 'Literature Agreement' column shows whether reported effect directions agreed to calculated effect directions.

Putative Annotation	*Molecular Subclass*	*Molecular Class*	*HMDB ID*	*PMID*	*Expected Effect Direction*	*Calculated Cliff's delta*	*Calculated Cliff's delta Size*	*Literature agreement*
Donepezil	Donepezil	AD Drug (Piperidines)	HMDB0005041		Increased	0.49717	large	Agree
Donepezil	Donepezil	AD Drug (Piperidines)	HMDB0005041		Increased	0.48862	large	Agree
DG(16:0/8:0/0:0)	Diacylglycerols	Glycerolipids	HMDB0093598	26402017	Increased	-0.19023	small	Disagree
DG(i-24:0/i-24:0/0:0)	Diacylglycerols	Glycerolipids	HMDB0094578	26402017	Increased	-0.20739	small	Disagree
TG(15:0/20:4/18:4)	Triglyceride	Glycerolipids	HMDB0043534	37879942	Decreased	-0.12990	negligible	Agree
LPC(O-18:1/0:0)_1	LysoPC	Glycerophospholipids	HMDB0013122	**30845751**	Decreased	0.24653	small	Disagree
LPC(O-18:1/0:0)_1	LysoPC	Glycerophospholipids	HMDB0013122	**30845751**	Decreased	0.26563	small	Disagree
LPC(O-18:1/0:0)_1	LysoPC	Glycerophospholipids	HMDB0013122	**30845751**	Decreased	0.25949	small	Disagree
LPC(O-18:1/0:0)_1	LysoPC	Glycerophospholipids	HMDB0013122	**30845751**	Decreased	0.23030	small	Disagree
LPC(O-18:1/0:0)_1	LysoPC	Glycerophospholipids	HMDB0013122	**30845751**	Decreased	0.28263	small	Disagree
LysoPC(20:0/0:0)	LysoPC	Glycerophospholipids	HMDB0010390	**30845751**	Decreased	-0.15203	small	Agree
PC(O-22:3/22:3)	Phosphatidylcholine	Glycerophospholipids	HMDB0013457	24041970	Decreased	-0.12190	negligible	Agree
PE(20:3-OH/22:4)	Phosphatidylethanolamine	Glycerophospholipids	HMDB0284125	**26811286**	Altered	0.17509	small	Agree
PE(20:3-OH/24:0)	Phosphatidylethanolamine	Glycerophospholipids	HMDB0284541	**26811286**	Altered	0.20568	small	Agree
PE(20:3-OH/24:1)	Phosphatidylethanolamine	Glycerophospholipids	HMDB0284645	**26811286**	Altered	0.20095	small	Agree
PE(24:1/20:3)	Phosphatidylethanolamine	Glycerophospholipids	HMDB0009761	**26811286**	Altered	0.21648	small	Agree
PE(24:1/20:3)	Phosphatidylethanolamine	Glycerophospholipids	HMDB0009761	**26811286**	Altered	0.20522	small	Agree
PE(P-18:1/24:1)	Phosphatidylethanolamine	Glycerophospholipids	HMDB0011462	**26811286**	Altered	-0.07275	negligible	Agree
PI(18:0/20:4)	Phosphatidylinositol	Glycerophospholipids	HMDB0009815	**30906529**	Decreased	-0.04449	negligible	Agree
Cer(18:2/20:3-OH)	Ceramide	Sphingolipid	HMDB0290094	36009503	Increased	0.36292	medium	Agree

Cer(20:1/18:1-O)	Ceramide	Sphingolipid	HMDB0290125	36009503	Increased	0.33846	medium	Agree
Cer(20:1/18:1-O)	Ceramide	Sphingolipid	HMDB0290125	36009503	Increased	0.46518	medium	Agree
Cer(20:1/18:1-O)	Ceramide	Sphingolipid	HMDB0290125	36009503	Increased	0.34871	medium	Agree
Cer(20:1/18:1-O)	Ceramide	Sphingolipid	HMDB0290125	36009503	Increased	0.37658	medium	Agree
Cer(20:1/18:1-O)	Ceramide	Sphingolipid	HMDB0290142	36009503	Increased	0.53987	large	Agree
Cer(20:1/18:1-O)	Ceramide	Sphingolipid	HMDB0290142	36009503	Increased	0.53498	large	Agree
Cer(20:1/18:1-O)	Ceramide	Sphingolipid	HMDB0290142	36009503	Increased	0.52263	large	Agree
Cer(20:1/18:1-O)	Ceramide	Sphingolipid	HMDB0290142	36009503	Increased	0.49266	large	Agree
CerP(18:1/12:0)	Ceramide phosphate	Sphingolipid	HMDB0010699	36525454	Increased	-0.14613	negligible	Disagree
CerP(18:1/12:0)	Ceramide phosphate	Sphingolipid	HMDB0010699	36525454	Increased	-0.15366	small	Disagree
CerP(18:1/12:0)	Ceramide phosphate	Sphingolipid	HMDB0010699	36525454	Increased	-0.14815	small	Disagree
CerP(18:1/12:0)	Ceramide phosphate	Sphingolipid	HMDB0010699	36525454	Increased	-0.15211	small	Disagree
CerP(18:1/12:0)	Ceramide phosphate	Sphingolipid	HMDB0010699	36525454	Increased	-0.15506	small	Disagree
CerP(18:1/12:0)	Ceramide phosphate	Sphingolipid	HMDB0010699	36525454	Increased	-0.13627	negligible	Disagree
CerP(18:1/12:0)	Ceramide phosphate	Sphingolipid	HMDB0010699	36525454	Increased	-0.14838	small	Disagree
SM(18:1/22:0)	Sphingomyelin	Sphingolipid	HMDB0012103	34397408	Decreased	-0.21997	small	Agree
3-O-Sulfogalactosylceramide (18:1/22:0)	Sulfatide	Sphingolipid	HMDB0012316	26542149	Increased	0.16119	small	Agree
3-O-Sulfogalactosylceramide (18:1/22:0)	Sulfatide	Sphingolipid	HMDB0012316	26542149	Increased	0.15630	small	Agree
3-O-Sulfogalactosylceramide (18:1/22:0)	Sulfatide	Sphingolipid	HMDB0012316	26542149	Increased	0.16849	small	Agree

3-O-Sulfogalactosylceramide (18:1/24:1)	Sulfatide	Sphingolipid	HMDB0012318	26542149	Increased	0.17540	small	Agree
3-O-Sulfogalactosylceramide (18:1/24:1)	Sulfatide	Sphingolipid	HMDB0012318	26542149	Increased	0.15584	small	Agree
CE(19:0)	Cholesteryl esters	Steroids and steroid derivatives	HMDB0006738	38488537	Increased	0.14178	negligible	Agree

2.3.1 Comparison to Non-integrative Models

Table 6. MAMSI Unique features. Features that were not present in non-integrative models. Values in 'Isotopologue Group', 'Isotopologue pattern', 'Structural Cluster', and 'Cross-assay link' correspond to values in SI Table 5. Both annotated and non-annotaed feature are present here.

Assay	*RT*	*m/z*	*Isotopologue group*	*Isotopologue pattern*	*Structural cluster*	*Cross-assay link*	*Annotation Kind*	*Putative Annotation*
HILIC +	138.81	470.2318					HMDB	Nefazodone
HILIC +	299.67	508.3765			2	2	HMDB | ROI	LPC(O-18:1/0:0)_1
HILIC +	37.23	567.4189			13	13		UNKNOWN
Lipid RPC -	370.54	887.6106	10	M+1	8		Extended	PE(24:1/20:3)
Lipid RPC -	186.16	509.3526						UNKNOWN
Lipid RPC -	378.21	922.6292	12	M	10		HMDB	3-O-Sulfogalactosylceramide (18:1/22:0)
Lipid RPC -	378.21	923.6322	12	M+1	10		Extended	3-O-Sulfogalactosylceramide (18:1/22:0)

Lipid RPC -	173.46	538.4185					HMDB	25(R)-Hydroxyprotopanaxadiol
Lipid RPC -	519.08	888.7213						UNKNOWN
Lipid RPC -	198.15	539.4307					HMDB	2-Hydroxydodecyl methacrylate
Lipid RPC -	275.33	551.4665					HMDB	1-Triacontanol
Lipid RPC +	138.19	509.3789	5	M+1	2		Extended	LPC(O-18:1/0:0)_1
Lipid RPC +	136.97	510.3793	5	M+2	2		Extended	LPC(O-18:1/0:0)_1
Lipid RPC +	524.52	831.7214					HMDB	DG(24:0/24:0/0:0)
Lipid RPC +	136.99	508.6063					HMDB	Dodec-6-enedioyl-CoA
Lipid RPC +	137.04	509.6703	6	M+1	3		Extended	anthocyanidin-5-O-glycosides

Table 7. Unique features to non-integrative single-block PLS models. Column 'Model' shows which assay one of the three single-block models used. 'AD Literature Association' column indicates whether given feature is associated with AD in literature and if so, a PMID is provided.

Model	*RT (sec)*	*m/z*	*Annotation Kind*	*Metabolite*	*Molecular Class*	*ID*	*AD Literature Association*	*PMID*
HILIC +	230.19	854.5674	HMDB	PC(22:5-O/P-18:1)	Glycerophospolipids	HMDB0289824	Associated	24041970
HILIC +	299.67	509.3797	ROI	LPC(O-18:1/0:0)_1	Phospholipid	HMDB0013122	Associated	30845751
HILIC +	226.82	868.6162	HMDB	PE(20:3-2OH/P-18:0)	Phospholipid	HMDB0285317	Associated	26811286
HILIC +	60.81	430.2434		UNKNOWN		Multiple		

HILIC +	230.19	870.5415		UNKNOWN		Multiple		
Lipid RPC -	477.17	847.6812	HMDB	TG(14:1/16:1/22:5)	Glycerolipids	HMDB0047928	Associated	37879942
Lipid RPC -	336.96	974.4923	HMDB	PC(22:5/22:5-O)	Glycerophospolipids	HMDB0288472	Associated	24041970
Lipid RPC -	52.15	351.1618	HMDB	N-Desethyl Sunitinib	Indoles and derivatives	HMDB0255114		
Lipid RPC -	368.26	722.5034	HMDB	PE(16:0/15:0)	Phospholipid	HMDB0008922	Associated	26811286
Lipid RPC -	368.16	720.4969	HMDB	PE(18:3-OH/P-18:1)	Phospholipid	HMDB0285569	Associated	26811286
Lipid RPC -	241.41	794.4971	HMDB	PE(20:2/5-iso)	Phospholipid	HMDB0262070	Associated	26811286
Lipid RPC -	343.46	774.537	HMDB	PE(P-18:1/20:2)	Phospholipid	HMDB0011448	Associated	26811286
Lipid RPC -	281.14	778.5144	HMDB	3-O-Sulfogalactosylceramide (18:1/16:0)	Sphingolipid	HMDB0012313	Associated	26542149
Lipid RPC +	403.02	861.6217	HMDB	PA(PGF1alpha/i-24:0)	Fatty Accids	HMDB0268550		
Lipid RPC +	496.06	666.6395	HMDB	DG(18:0/21:0/0:0)	Glycerolipids	HMDB0093981	Associated	26402017
Lipid RPC +	276.69	635.4835	HMDB	DG(18:1-2OH/0:0/15:0)	Glycerolipids	HMDB0299044	Associated	26402017
Lipid RPC +	495.91	833.7018	HMDB	TG(14:0/20:3/18:3)	Glycerolipids	HMDB0042437	Associated	37879942
Lipid RPC +	670.55	888.8167	HMDB	TG(15:0/O-18:0/16:1)	Glycerolipids	HMDB0043808	Associated	37879942
Lipid RPC +	517.45	537.0248	HMDB	Norepinephrine sulfate	Organic sulfuric acids and derivatives	HMDB0002062		
Lipid RPC +	517.87	268.2147	Extended (Dimer)	Norepinephrine sulfate	Organic sulfuric acids and derivatives	HMDB0002062		
Lipid RPC +	654.78	879.1947	HMDB	Darglitazon	Organooxygen compounds	HMDB0250869		

Lipid RPC +	646.27	863.0888	HMDB	lamotrigine-2-N-glucuronide	Organooxygen compounds	HMDB0061103		
Lipid RPC +	656.71	1028.7369	HMDB	N-Oleylcytosine arabinoside	Pyrimidine nucleosides	HMDB0255284		
Lipid RPC +	529.68	669.6578	Extended (Isotoplogue)	Cer(18:1/23:0)	Sphingolipid	HMDB0000950	Associated	36009503
Lipid RPC +	529.75	668.6548	HMDB	Cer(18:1/23:0)	Sphingolipid	HMDB0000950	Associated	36009503
Lipid RPC +	276.52	536.5024	HMDB	Cer(18:2/16:0)	Sphingolipid	HMDB0240686	Associated	36009503
Lipid RPC +	261.61	568.4263	HMDB	CerP(18:1/12:0)	Sphingolipid	HMDB0242380	Associated	36525454
Lipid RPC +	392.79	811.9733	HMDB	Ganglioside GD3 (18:0/26:1)	Sphingolipid	HMDB0011870		
Lipid RPC +	161.79	503.3167		UNKNOWN		None		
Lipid RPC +	230.89	549.409		UNKNOWN		Multiple		
Lipid RPC +	259.4	552.4272		UNKNOWN		Multiple		
Lipid RPC +	670.79	605.1619		UNKNOWN		Multiple		

References

[1] A. Baum and L. Vermue. "Multiblock Partial Least Squares Package." https://mbpls.readthedocs.io/en/latest/index.html (accessed Oct, 2022).

[2] M. Farrés, S. Platikanov, S. Tsakovski, and R. Tauler, "Comparison of the variable importance in projection (VIP) and of the selectivity ratio (SR) methods for variable selection and interpretation," *J. Chemom.,* vol. 29, no. 10, pp. 528-536, 2015, doi: 10.1002/cem.2736.

[3] C. Wieder *et al.*, "PathIntegrate: Multivariate modelling approaches for pathway-based multi-omics data integration," *PLOS Comput. Biol.,* vol. 20, no. 3, p. e1011814, Mar 2024, doi: 10.1371/journal.pcbi.1011814.

[4] T. Kind. "Mass Spectrometry Adduct Calculator." https://fiehnlab.ucdavis.edu/staff/kind/metabolomics/ms-adduct-calculator/ (accessed April 14, 2024).

[5] Waters. "Waters Knowledge Base: What are common adducts in ESI mass spectrometry?" https://support.waters.com/KB_Chem/Other/WKB67428_What_are_common_adducts_in_ESI_Mass_Spectrometry (accessed Apr 26, 2024).

[6] National Phenome Centre, D. a. R. Metabolism, and L. W. N. Imperial College London, United Kingdom. "The National Phenome Centre's Open Platform for LC-MS-Based Metabolomics." GitHub.com. https://github.com/phenomecentre/npc-open-lcms (accessed April 30, 2024).

[7] A. M. Wolfer *et al.*, "peakPantheR, an R package for large-scale targeted extraction and integration of annotated metabolic features in LC–MS profiling datasets," *Bioinformatics,* vol. 37, no. 24, pp. 4886-4888, 2021, doi: 10.1093/bioinformatics/btab433.
